# Animal Models of Craving: A Roundtable Discussion

**Published:** 1999

**Authors:** Barbara Breen Vann

**Affiliations:** Barbara Breen Vann, managing editor of Alcohol Research & Health, and Lori A. Wolfgang, M.A., a science editor of Alcohol Research & Health, coordinated the panel discussion

**Keywords:** AOD (alcohol and other drug) craving, animal model, animal behavior, AOD use behavior, theory of AODU (alcohol and other drug use), operant conditioning, motivation, alcohol cue, AODD (alcohol and other drug dependence) relapse, AOD withdrawal syndrome, disorder definition, laboratory study, interview

## Abstract

Five experts respected for their work in the development of animal models of alcohol craving offer their perspectives in a roundtable discussion format. The panel members discuss the various definitions and theories of alcohol craving and the benefits and limitations of using animal models to study alcohol craving in humans. Animal models have helped further the understanding of craving by providing information about behavior associated with craving. Animal models do have limitations, however. The fact that animals cannot “talk” about their feelings poses difficulties for researchers seeking to map an animal analog of craving onto the human experience.

The use of animals to model humans has long been an integral part of medical and scientific research into human functions and conditions. Research using animals has led to many important medical discoveries in the past century, from the use of depancreatized dogs in 1921 to study the effects of insulin to the recent mapping and sequencing of rat, mouse, and fruit fly genomes to better understand human genetic makeup.

Likewise, the field of alcohol research has benefited from a number of discoveries that were first identified using animal models. The development of animal models for alcoholism began in the 1940s. Since that time, rats and monkeys have been used to model different drinking behaviors and to study how alcohol damages different bodily organs. Animal models also have helped scientists to analyze the changes in brain chemistry that occur when alcohol is consumed. Perhaps most promising, genetically altered animal models are proving to be valuable in the search for genes that may be involved in the development of alcoholism.

Whereas the use of animal models to study the physiological effects of alcohol on tissue and organs has been fairly straightforward, using animal models to assess psychological effects raises questions. How do scientists measure “craving,” the uncontrollable desire for alcohol that is associated with alcohol dependence?

*Alcohol Research & Health (AR&H)* asked several renowned scientists currently working in the field to share their views on animal models of human alcohol craving. Our panel of experts includes the following:

- *George F. Koob, Ph.D.*—professor in the Department of Neuropharmacology at The Scripps Research Institute, La Jolla, California
- *Friedbert Weiss, Ph.D.*—associate professor in the Department of Neuropharmacology at The Scripps Research Institute, La Jolla, California
- *Stephen T. Tiffany, Ph.D.*—professor in the Department of Psychological Sciences at Purdue University, West Lafayette, Indiana
- *Walter Zieglgansberger, Ph.D.*—Head of Clinical Neuropharmacology at the Max Planck Institute of Psychiatry, Munich, Germany
- *Rainer Spanagel, Ph.D.*—Head of Drug Abuse Research at the Max Planck Institute of Psychiatry, Munich, Germany

***AR&H: What would you say is the greatest advantage to using animal models to study alcohol craving?***

***Tiffany:*** Without question, animal studies have taught us and can continue to teach us a great deal about craving. Most importantly, animal research offers a rich source of ideas regarding the fundamental nature of drug craving. Indeed, almost all of the major conceptualizations of craving developed over the past 50 years originated in the animal laboratory.

The link between animal research and craving is obvious when you consider major craving theories like Abraham Wikler’s conditioned-withdrawal model, Shepherd Siegel’s compensatory-response model, or Jane Stewart’s incentive-motivational model. Wikler’s model proposed that situations paired with drug withdrawal (e.g., being in an alcohol detoxification ward in a treatment clinic) become conditioned stimuli that trigger conditioned withdrawal reactions. These reactions might include sweating, racing heart, and nausea. The theory proposed that in humans, these conditioned reactions would trigger craving, which would trigger drug use. Siegel’s model is very similar to Wikler’s. The major difference is that Siegel proposed that situations paired with drug use (e.g., being with other drug users), rather than drug withdrawal, could produce conditioned withdrawal reactions and craving. The craving model developed by Stewart and colleagues emphasized the reinforcing effects of substance use in the generation of craving. In this model, situations associated with substance use (e.g., the sight and smell of alcohol) become conditioned reinforcers. These reinforcers activate positive motivational states that produce craving and drug-seeking behaviors. Less obvious, but equally important, is the influence of concepts borrowed from animal learning theory in the development of social-cognitive models of craving. For example, G. Alan Marlatt has hypothesized that alcohol craving represents a person’s expectations about the positive effects of alcohol. The idea that expectancies about outcomes strongly influence behavior was taken directly from animal models of learning.

***AR&H: One of the most puzzling questions is, “What is craving?” How does your lab define craving when dealing with animal models?***

***Zieglgansberger and Spanagel:*** There are opposing views regarding the term craving. Does it describe a physiological, subjective, or behavioral state? Indeed, does craving play a role in addictive behavior and relapse? When defining craving within the framework of the incentive motivational theories of behavior, it can be described as the incentive motivating a person (or animal) to consume a psychoactive drug. This definition makes it possible to measure craving in laboratory animals, and thus to study the factors that influence craving and the significance of craving for relapse. However, such a definition disregards the subjective dimension of craving, which is difficult if not impossible to measure in laboratory animals.

***Koob:*** In fact there is no such thing as a single animal model of craving. There can be animal models of the various aspects, syndromes, and domains associated with craving, however.

***Weiss:*** I agree with Dr. Koob. Like all animal models of human behavior or pathology, animal models of craving can provide valuable information about aspects of the construct they are intended to model but, at the same time, have clear limitations. Not only is the definition of craving and its significance for alcohol abuse in humans disputed, but craving is obviously a hypothetical construct and cannot be studied directly in animals. Instead, we have to rely on operationally defined behavioral responses that we interpret as reflecting craving. Whether or not the behavior observed is in fact motivated by an internal state such as “desire” or “craving” cannot be known. For example, as stated by Drs. Zieglgansberger and Spanagel, there continues to be debate as to whether relapse evoked by drug- or alcohol-related environmental cues involves cue-induced craving or simply automatic responses that are unrelated to conscious desire [see also Miller and Gold 1994; Tiffany and Carter 1998].

***Tiffany:*** As suggested in the comments by Drs. Koob, Weiss, and Zieglgansberger and Spanagel, we really never study craving in animals; we use animals to study *theories of craving*. For me, the answer to the question, “What is craving or how do you study craving in animals?” is determined by your model of craving. For example, if you propose that craving is a conditioned alcohol withdrawal response, then you can study craving in animals by conducting research on conditioned-withdrawal effects. Or you might propose that craving represents an expectancy that alcohol use will produce positive effects. There are certainly animal paradigms for investigating those kinds of expectancies. I suspect that any theory of craving might describe aspects of hypothesized craving processes that could be examined in animals. Certainly, some theories are more useful than others because they permit very precise predictions about how craving operates and how it is expressed in behavior. Such theories serve as powerful tools for designing animal or human craving studies. Other craving theories are less explicit and are not particularly helpful for guiding craving research, be it animal or human.

***AR&H: Considering the numerous limitations of animal models, what then is the value of using laboratory animals to model human behavior?***

***Tiffany:*** Studies with animals allow us to conduct experiments that would simply not be possible in human subjects. For example, we cannot, either ethically or practically, make someone dependent on alcohol so as to discover how craving processes unfold in the developing alcoholic. Further, we cannot use human subjects in experiments involving important pharmacological or neurobiological manipulations, although such experiments with animals may reveal much about the biological control of craving. Finally, there are certain biological processes that may be relevant to craving, such as brain cell function or brain cell communication that cannot, at present, be easily measured in humans but can be measured in animals.

***Weiss:*** I agree with Dr. Tiffany that animal models of craving are valuable. They can provide an effective and inexpensive tool to study alcohol-seeking behavior under controlled laboratory conditions and without the ethical constraints associated with human addiction research. In addition, of course, studies of the neurobiological basis of these behaviors or medication development and testing can only be done in animals.

***Koob:*** That’s correct. Proper validation of an animal model’s ability to predict human outcomes should eliminate any “gap” between what is learned in animal studies and what can be applied to humans. For example, the elevated plus maze (see figure, p. 235) is a validated animal model of anxiety and has provided substantial insight into the neuropharmacology of anxiety and the development of anti-anxiety drugs, but no one would argue that a rat on the plus maze is “anxious.”

***Weiss:*** The bottom line is that animal models do not necessarily have to be fully analogous or isomorphic (i.e., alike in form or shape) with respect to the human condition they model. Thus, a “simple” behavioral model, such as the plus maze mentioned by Dr. Koob, can be very useful for studying anxiety in humans provided the model has predictive validity, that is, the potential to lead to accurate predictions about anxiety and its treatment in humans.

***AR&H: Dr. Weiss, your lab is currently using an animal model that is proving highly useful. Would you briefly discuss what this model entails?***

***Weiss:*** Our laboratory uses a model of alcohol-seeking behavior in which rats press a lever to receive alcohol. The rats first learn that alcohol is available only in the presence of a particular olfactory cue, such as the smell of anise, but not in the presence of a different odor. We then remove both the alcohol and the cues until the rats stop responding at the lever that previously produced alcohol, a procedure that takes 2 to 3 weeks. When the alcohol-associated cue is then reintroduced, but without alcohol being available, the rats immediately resume responding at the lever. The rats do not respond, however, when the cue that was not paired with alcohol is reintroduced. When the rats are given the anticraving drug naltrexone, they stop seeking alcohol in response to the alcohol cues. Since studies with naltrexone in humans indicate that this drug can reduce alcohol craving, this model has predictive validity, although, strictly speaking, as a model of relapse rather than craving per se.

Environmental stimuli that have been repeatedly paired with drinking over the course of a person’s history of alcohol use can elicit strong cravings for alcohol such that this model and its use of alcohol cues may in fact qualify as an animal analog of craving. I prefer to think of it as a model of relapse, however, because what we are actually measuring is the recovery of a behavioral response that had been extinguished, and we cannot be sure what the underlying processes are.

***AR&H: What would you say is the key strength of this model?***

***Weiss:*** One of the primary strengths of this model is that it allows us to study the mechanisms involved in the motivation to engage in alcohol-seeking behavior independently of the processes that control the reinforcing actions of alcohol consumption per se. It can also be used to study the persistence of the motivating effects of alcohol cues over time as well as behavioral or pharmacological interventions that may accelerate the extinction of alcohol seeking in response to alcohol cues. Therefore, the model is applicable to a range of questions surrounding the issues of craving and relapse. We are currently using this model to pinpoint neural circuitries and neurochemical systems that mediate this type of cue-induced alcohol-seeking behavior as well as to identify pharmacological agents that reduce this behavior.

**Figure:**
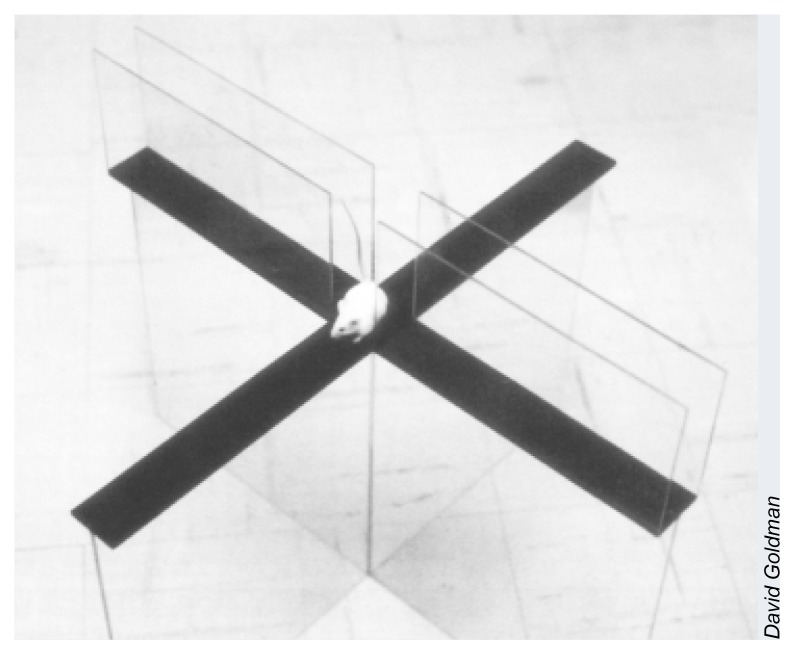
A plus maze is used to study the anxiolytic effect of alcohol. Although rodents normally avoid open sections of the maze, they run freely into those areas after drinking alcohol. This behavior suggests that alcohol may lessen the anxiety that the animals experience in open spaces.

***AR&H: Drs. Zieglgansberger and Spanagel, you mentioned earlier that your model defines craving as the incentive motivating a person (or animal) to consume a psychoactive drug. Would you describe this model in more detail?***

***Zieglgansberger and Spanagel:*** In our model, several months of alcohol availability (and voluntary alcohol consumption) are followed by a period of alcohol deprivation (i.e., a withdrawal phase). When alcohol is subsequently made available, the animals increase their alcohol consumption and preference for alcohol (i.e., they demonstrate a preference for alcohol over water) and exhibit changes in their alcohol-intake patterns [Spanagel and Hölter 1999]. This increase in alcohol consumption and preference is known as an alcohol deprivation effect and is also observed in humans. For example, the animals may consume large amounts of highly concentrated alcohol solutions even at inappropriate times (e.g., during the inactive light phases when drinking activity is usually low). We have also observed such an alcohol deprivation effect under operant conditions. After depriving one group of animals of alcohol for 2 weeks while providing continuous alcohol access to a comparison group, we placed the animals in testing chambers for 23-hour sessions during which the animals could access both a 20 percent alcohol solution and water. The animals that had been alcohol-deprived consumed significantly more alcohol and demonstrated significantly greater preference for alcohol during the testing sessions compared with animals not deprived of alcohol [Hölter et al. 1997].

The introduction of a progressive ratio task, in which the animal must perform progressively more work (e.g., increasing number of lever presses) to repeatedly receive the reinforcer (e.g., a drop of alcohol), further demonstrates this high motivation to drink alcohol following a period of deprivation. For example, following alcohol deprivation, animals will continue to work for alcohol significantly longer than they would before the alcohol deprivation period. Thus, alcohol-deprived animals are more willing to work for alcohol (i.e., they have a higher motivation) than animals that have not been alcohol deprived [Spanagel and Hölter 1999]. Furthermore, alcohol consumption after a period of alcohol deprivation is not affected by the addition of quinine to alcohol solutions (to make alcohol unpalatable) or the additional presentation of a highly palatable sucrose solution [Spanagel et al. 1996; Spanagel and Hölter 1999].

These findings demonstrate a non-nutritional component of alcohol consumption and pharmacologically motivated drinking behavior in rats exposed to extended periods of alcohol availability. We conclude that alcohol consumption after a period of alcohol deprivation is characterized by an uncontrolled motivation to consume the drug. This conclusion is fully compatible with our initial operating definition of craving. However, the measurement of an alcohol deprivation effect in long-term alcohol-drinking rats accesses only a behavioral outcome; it does not tell us anything about a subjective state associated with the alcohol deprivation effect. Nevertheless, the fact that clinically effective anticraving and antirelapse compounds reduce the alcohol deprivation effect in our animals lends predictive value to this animal model for the development of new and better drugs for the treatment of alcoholism [Spanagel et al. 1996; Hölter et al. 1997; Spanagel and Zieglgansberger 1997; Hölter and Spanagel 1999]. However, there are clear limitations on using such an animal model to explain the phenomenon of craving and the relationship of craving and relapse behavior.

***AR&H: One of the challenges of using animal models to simulate human behavior is extrapolating the data and applying it to humans. How do you determine whether the findings in animals actually extend to humans?***

***Weiss:*** Again, because animal models of craving depend on measures of behavioral responses rather than internal states, the concerns and limitations raised earlier apply. However, as previously pointed out, animal models do not have to be isomorphic to the condition they model to serve as potentially useful models for medication development. There is a need to better understand the craving phenomena associated with different stages of the addictive cycle in humans in order to establish effective animal models or to effectively apply findings from animal models. Craving is likely to be a multidimensional phenomenon [e.g., Markou et al. 1993]. For example, craving has been associated with acute withdrawal, protracted abstinence, stress, environmental cues, and alcohol consumption itself (i.e., the priming effect, in which drinking a small amount of alcohol actually leads to craving for more). It seems clear, for example, that craving experienced during acute withdrawal is different from craving that occurs long after detoxification and abstinence. But what about craving elicited by alcohol cues, by stress, or by a drink of alcohol itself? Are these states of craving subjectively identical or different, and do they have similar or different effects in terms of their likelihood to lead to drinking or relapse? A better understanding of the range of conditions that lead to cravings and their respective roles in the resumption or exacerbation of drinking may help guide and focus the development of animal models of craving. This may help establish, for example, whether alcohol-seeking behaviors induced by different “triggers” for craving have a common or distinct neurobiological basis and, consequently, whether the prevention of alcohol abuse or relapse associated with these conditions would require similar or different pharmacotherapeutic strategies.

***Tiffany:*** Despite their considerable promise, animal models of craving can be limited in two ways. First, one of the prominent distinctions between animal and human craving is that humans can talk about their levels of craving and animals cannot. Some addiction scientists assume that verbal or language-based descriptions of craving directly and uniquely define the essence of craving. From this perspective, animal models could never capture craving in humans.

Even if you do not adopt the view that craving can only be measured through language-based descriptions, the fact that animals cannot talk poses difficulties for researchers who want to map an animal analog of craving onto the human experience. These problems could be overcome if we could identify a response that is strongly associated with addicts’ descriptions of their craving. For example, it would make things easy if a physiological measure such as change in heart rate could be used interchangeably with craving measures. Such a measure could be used in animal research as a substitute for craving reports. Unfortunately, researchers have not yet identified any measure, physiological or otherwise, that gives us even a remote substitute for reports of craving by addicts.

Many animal models of craving also are limited in that they use concepts of learning and motivation that date from the 1950s and 1960s. In many cases, these concepts are no longer widely accepted in psychology and have been replaced by distinctly different theories (see Tiffany 1995). One goal of future research should be to represent modern theories of animal learning, memory, and motivation in animal models of craving.
